# Effect of Dietary Cholesterol and Cholesterol Oxides on Blood Cholesterol, Lipids, and the Development of Atherosclerosis in Rabbits

**DOI:** 10.3390/ijms140612593

**Published:** 2013-06-17

**Authors:** Sun Jin Hur, Byungrok Min, Ki Chang Nam, Eun Joo Lee, Dong Uk Ahn

**Affiliations:** 1 Department of Bioresources and Food Science, Konkuk University, 120 Neungdong-ro, Gwangjin-gu, Seoul 143-701, Korea; E-Mail: sjhur@konkuk.ac.kr; 2 Food Science and Technology Program, Department of Agriculture, Food, and Resource Science, University Maryland Eastern Shore, Princess Anne, MD 21853, USA; E-Mail: bmin@umes.edu; 3 Department of Animal Science and Technology, Sunchon National University, Sunchon 540-742, Korea; E-Mail: kichang@scnu.kr; 4 Department of Food and Nutrition, University of Wisconsin-Stout, Menomonie, WI 54751, USA; E-Mail: leeeu@uwstout.edu; 5 Department of Animal Science, College of Agriculture, Iowa State University, Ames, IA 50011, USA; 6 WCU Biomodulation, Department of Agricultural Biotechnology, Seoul National University, 1 Gwanak-ro, Gwanak-gu, Seoul 151-921, Korea

**Keywords:** dietary cholesterol, cholesterol oxides, atherosclerosis, serum cholesterol, HDL

## Abstract

Two studies were conducted to determine the effects of dietary cholesterol (CHO) and cholesterol oxides (COPs) on the development of atherosclerosis and the changes in fatty acid and blood characteristics in rabbits. In the first study, forty male New Zealand white rabbits were divided into 5 groups and fed commercial rabbit chow with no added CHO or COPs, 1 g CHO, 0.9 g CHO + 0.1 g COPs, 0.8 g CHO + 0.2 g COPs, or 0.5 g CHO + 0.5 g COPs per kg diet. In the second study, 24 male New Zealand White rabbits were divided into 3 groups and fed a diet containing 2 g CHO, 1.6 g CHO + 0.4 g COPs, or 1.2 g CHO + 0.8 g COPs per kg diet. All diets induced atherosclerotic lesions in the rabbits’ ascending thoracic aorta. The serum CHO and triglyceride levels (*p <* 0.05) increased significantly with the increased levels of CHO in the diets. Dietary CHO or COPs did not influence high-density lipoprotein CHO levels. The ratio of saturated fatty acid to unsaturated fatty acid increased as the level of dietary CHO and COPs increased.

## 1. Introduction

Cholesterol (CHO) is an important component in the human body but high levels of CHO is considered a major risk factor related to the development of atherosclerosis and coronary heart disease (CHD). The literature on atherosclerosis and CHD is dominated by two compelling hypotheses: the “lipid hypothesis” and the “response-to-injury hypothesis.” CHO plays a pivotal role in the lipid hypothesis, whereas in the response-to-injury theory, cell damage by oxidized CHO initiates atherosclerosis and CHD [1]. The lipid hypothesis suggests that atherosclerosis and CHD are caused by hypercholesterolemia-induced lipid deposition in the vessel wall. Over the last 15 years, evidence has accumulated to suggest that the major component that causes CHD is not the natural, but the oxidized form of CHO. Diets high in CHO and saturated fat have been shown to promote the development of atherosclerosis [2]. Higley *et al.* [3] reported that in rabbits, CHO is more atherogenic than COPs alone or COPs in combination with CHO. Dornas *et al.* [4] demonstrated that feeding large doses of CHO to rabbits results in hypercholesterolemia that induces atherosclerotic lesions in the aorta. Ahmad [5] also reported that hypercholesterolemia-induced lipid deposition in the blood vessel walls is the major cause of atherosclerosis and CHD.

The etiology of cardiovascular disease is complex and multifactorial, but there is substantial evidence that oxidized lipoproteins play an important role in atherosclerosis. Many COPs are cytotoxic, atherogenic, mutagenic, and carcinogenic. Several recent studies have demonstrated COP involvement in the rapid progression of CHD and atherosclerosis in animals. Poli *et al.* [6] reported that COPs injure endothelial cells, causing atherosclerosis. In addition, several observations suggested that oxidized lipoproteins were important in the development of atherosclerosis [7]. Li *et al.* [8] and Sevanian *et al.* [9] reported that COPs cause endothelial cell damage, which initiates the complex series of pathological changes that ultimately lead to plaque formation. The oxidation of low-density lipoprotein (LDL) has been strongly implicated in the pathogenesis of atherosclerosis [8], and lipid oxidation products (LOPs) can oxidize CHO. It is difficult to say whether COPs or natural CHO is more important in the development of atherosclerosis and CHD. Therefore, the objectives of this study were to determine the effects of dietary CHO and COPs on the development of atherosclerotic lesions, fatty acid, and blood characteristics in rabbits.

## 2. Results

### 2.1. Histopathology

Histopathological results are summarized in Table 1. Histologically, aortic lesions had very large, pale, vacuolar, lipid-filled macrophages. The lipid-filled macrophages of the plaques were strongly positive for oil-red-O but did not stain blue for glycoproteins/glycolipids at an acidic pH or with the periodic acid-Schiff (PAS) procedure. The oil-red-O stain indicated the presence of lipids in the cytoplasm of smooth muscle cells of the aortic media.

The lesion severity was based on the histopathological features of the samples: 0, no abnormalities detected; 1+, focal aggregations of 4–8 foam cells in the tunica intima; 2+, focal aggregations of >8 foam cells with small lipid accumulations; 3+, a thick lipid layer and low numbers of foam cells in the tunica intima extending part way around the blood vessels; and 4+, a diffused thick lipid layer with moderate numbers of foam cells infiltrating the tunica intima and extending around the circumference of the blood vessel and bulging into the lumen.

Three rabbits that were fed 0 g CHO did not develop atherosclerotic lesions in the ascending thoracic aorta or the coronary artery. In all other rabbits in both studies, atherosclerosis was seen at varying levels of severity that was characterized by the deposition of lipids and infiltration of foam cells and macrophages. In all rabbits examined, lesions in the ascending thoracic aorta were confined to the tunica intima, and lesions in the coronary artery were more commonly found adjacent to the base of the ascending aorta or adjacent to the aortic or pulmonary valve leaflets. The remaining rabbits in Study 1 developed mild focal aggregations of 4–8 foam cells in the tunica intima (lesions of 1+ severity). No different lesions were seen between rabbits necropsied at 6 and 12 weeks after the beginning of the feeding trial. In contrast, most rabbits in Study 2 developed more severe atherosclerotic lesions than those in Study 1. Lesions were also more severe in rabbits necropsied at 12 weeks after the feeding trial compared to those necropsied at 6 weeks. Lesions in the ascending thoracic aorta of the rabbits in Study 2 were confined to the tunica intima, and lesions in the coronary artery were more commonly found adjacent to the base of the ascending aorta or adjacent to the aortic or pulmonary valve leaflets, as seen in the rabbits in Study 1.

In rabbits necropsied at 6 weeks after the beginning of feeding 2 g CHO, 3 out of 4 rabbits had lesions of 2+ severity or greater in both the ascending thoracic aorta and the coronary artery, and the one remaining rabbit had 1+ lesions in both the ascending thoracic aorta and the coronary artery. In the 1.6 g CHO + 0.4 g COPs, 2 of 4 rabbits had lesions of 2+ severity or greater in the ascending thoracic aorta and the coronary artery, and 2 rabbits had lesions of 1+ severity. In the 1.2 g CHO + 0.8 g COPs group, 2 of 4 rabbits had at least 2+ severity in the ascending thoracic aorta and 2 had 1+ lesions. In the 1.6 g CHO + 0.4 g COPs, 3 of 4 rabbits had lesions of 2+ severity or greater in the ascending thoracic aorta and the coronary artery. In the 1.2 g CHO + 0.8 g COPs group, 1 rabbit had a 3+ lesion in the ascending thoracic aorta and a 2+ lesion in the coronary artery. Another rabbit in this group had a 2+ lesion in the ascending thoracic aorta. The remaining 2 rabbits had mild lesions in both the ascending thoracic aorta and the coronary artery.

### 2.2. Cholesterol Contents

Serum total CHO levels increased (*p <* 0.05) markedly after 2 weeks of dietary CHO treatments and reached their maximum levels after 10 or 12 weeks (Table 2). The mean serum CHO level after 12 weeks of the feeding trial was 28.57 μmol/L for control rabbits and ranged from 115.9 to 580.5 μmol/L for the CHO and CHO + COPs groups. The rabbits fed high-CHO diets had high serum CHO levels, rabbits receiving 2 g dietary CHO showed the highest serum CHO levels, and the control (0 g) group showed the lowest serum CHO levels among the treatment groups. Dietary CHO levels significantly affected serum CHO levels, but the effect was less than that by dietary COPs in the rabbits.

### 2.3. Triglyceride Contents

The triglycerides (TG) levels in serum increased gradually in all rabbits as the feeding time increased (Table 3). However, the rabbits fed high CHO diets had higher serum CHO levels than those fed low-CHO diets. The changes of TG levels among dietary CHO groups showed similar trends as in serum CHO, and rabbits receiving 2 g CHO had significantly (*p <* 0.05) higher TG levels than the other diet groups. The control (0 g) rabbits had similar TG levels to the 0.9 g CHO + 0.1 g COPs group after 12 weeks of the feeding trial.

### 2.4. LDL-Cholesterol Contents

Table 4 shows the effect of dietary CHO and COPs on serum LDL-CHO levels. Serum LDL-CHO levels increased as the total dietary CHO increased. The LDL-CHO levels were markedly increased after 2 weeks of CHO feeding and reached their maximum at 10 weeks. The LDL-CHO levels in serum were significantly (*p <* 0.05) higher in rabbits fed diets containing 2 g total CHO (CHO + COPs) (Study 2) than those fed 1 g total CHO (Study 1). After 12 weeks of the feeding trial, the 2 g CHO and 1.6 g CHO + 0.4 g COPs groups had significantly (*p <* 0.05) higher LDL-CHO levels than those fed low CHO diets (1 g total CHO), which showed a similar trend in the development of atherosclerotic lesions in the ascending thoracic aorta.

### 2.5. HDL-Cholesterol Contents

Serum HDL-CHO level of all rabbits gradually increased (*p <* 0.05) as the feeding periods increased (Table 5). At 4 and 6 weeks of the feeding trial, the serum CHO level in rabbits receiving 2 g CHO was significantly (*p <* 0.05) higher than that of the other groups, but did not differ after this period.

### 2.6. Fatty Acid Composition

Dietary CHO and COPs markedly influenced the fatty acid composition of erythrocyte (RBC) membranes (Table 6). Dietary CHO and COPs increased monounsaturated fatty acid (MUFA) and polyunsaturated fatty acid (PUFA) levels and decreased saturated fatty acid (SFA) levels. Dietary COPs had a stronger effect than CHO on the fatty acid composition of RBCs. The percentage of palmitic acid and stearic acid levels decreased (*p <* 0.05), but those of oleic, linoleic, and linolenic acids in RBC membranes increased with dietary CHO and COPs. The level of arachidonic acid also increased significantly (*p <* 0.05) with the dietary CHO and COPs intake but did not have much impact on the ratio of SFA:MUFA:PUFA because the levels of arachidonic acid were very small.

## 3. Discussion

Previous research [9,10] suggested that the natural form of CHO was neither atherogenic nor angiotoxic despite being able to induce hypercholesterolemia. However, hypercholesterolemia is widely recognized as an important risk factor for atherosclerosis development [11]. Over the last 15 years, evidence has accumulated to suggest that CHO, not in its natural, but oxidized form, is the major factor for CHD. However, this study showed that not only dietary COPs, but also high CHO levels are associated with the increased risk of atherosclerosis in rabbits. This may be because the high levels of natural CHO are oxidized during digestion or metabolism. In the stomach, CHO or lipids are exposed to a highly acidic environment (pH 1–3), and they may be subjected to enzyme hydrolysis (e.g., protease or gastric lipase). In the small intestine, the partially hydrolyzed CHO is mixed with digestive juices (e.g., CHO esterase), and the pH reaches close to neutral due to the mixing of the chime with alkali digestive juice (e.g., bile salt or sodium bicarbonate), which contributes to the oxidation of CHO.

This may explain why both dietary COPs and high natural CHO levels can be associated with the increased risk of atherosclerosis in rabbits. In a previous study, Staprans *et al.* [12] also demonstrated that feeding nonoxidized (control) or oxidized CHO markedly increased serum CHO concentrations in rabbits after 2 weeks. Indeed, the relationship between serum CHO levels and atherosclerosis has been studied for more than 80 years, and the hypercholesterolemia-induced deposition of lipid in vessel walls has been considered the major cause of atherosclerosis and CHD [5]. Yang *et al.* [13] also demonstrated that feeding a high-CHO diet to rabbits for 10 weeks resulted in marked hypercholesterolemia and the development of atherosclerosis. Thus, we assume that the increased risk of atherosclerosis may depend on the amount of dietary COPs and CHO and may not depend on the form of CHO (oxidized or natural form).

Paik and Blair [14] reported the inverse relationship between HDL-CHO, and the risk of atherosclerosis and coronary artery diseases (CAD) is well established: generally, 1 mg/dL increment in HDL-CHO is associated with a 2% decrease in CAD risk in men and a 3% decrease in women. However, our results showed that dietary CHO and COPs did not significantly alter HDL-CHO levels at the end of the feeding trial, but significantly increased atherosclerotic lesions in rabbits. Paik and Blair [14] reported that TG-rich lipoproteins are not considered atherogenic, but they are related to the metabolism of HDL-CHO and are indirectly related to CHD. However, serum TG, LDL-CHO, and total CHO levels increased with the increase of dietary CHO intake and were directly related to atherogenic lesions.

In general, serum LDL-CHO levels correlated well with the quantity of oxidized lipids in the diet, and feeding CHO and COPs correlated with increased serum LDL-CHO levels. Grundy [15] and Zandberg *et al.* [16] also reported that dietary CHO suppresses the activity of LDL receptors by increasing the serum CHO levels. Therefore, high serum CHO and LDL levels might have increased the deposition of CHO in the vessel wall and promoted the development of atherosclerosis, which is similar to our results. However, Van Craeyveld *et al.* [17] reported that the atherogenic particle in cholesterol fed rabbits is not simply LDL but predominantly β-VLDL and intermediate density lipoproteins (IDL). This may be due to the fact that not only LDL, but also VLDL, are the vehicles to supply cholesterol throughout the body in order to maintain cell viability. Moreover, LDL, VLDL and IDL contain high amounts of cholesterol. Van Craeyveld *et al.* [17] also reported that the relative atherogenicity of VLDL and LDL is dependent on the topographic site, and correlation of the extent of atherosclerosis between different sites is dependent on the type of hyperlipidemia [18]. It may be due to that oxidative stress is different in atherosclerosis-predisposed regions, and atherosclerosis is an inflammatory disease in which various cytokines play a significant role in the progression of vascular lesions [19].

In the present study, dietary CHO and COPs increased MUFA and PUFA and decreased SFA. The percentage of palmitic acid and stearic acid levels decreased (*p <* 0.05), but those of oleic, linoleic, and linolenic acids in the RBC membrane increased with dietary CHO and COPs. Schouten *et al.* [20] reported that CHO significantly reduced stearic acid level but increased linoleic acid level in the erythrocytes of rabbits. They postulated that the increase in CHO/phospholipid ratio in erythrocytes is compensated by a decrease in palmitic acid (C18:0) and an increase in linoleic acid (C18:2n6) mainly because CHO feeding increased Δ^9^-desaturase activity and decreased Δ^6^- and Δ^5^-desaturase activities. Iuliano *et al.* [21] also reported that oxysterols were closely related to the altered fatty acid profile.

Thus, we assume that dietary COPs and CHO can influence the increases in unsaturated fatty acid levels. In general, unsaturated fatty acids are molecules with an unsaturated or double bond; therefore, they are prone to oxidation. They are also sensitive to free-radical oxidation by diatomic molecular oxygen, and oxidized fatty acids play a major role in the action of oxidized LDL. Rudel *et al.* [22] also reported that LDL particles enriched in polyunsaturated fatty acids are more easily oxidized *in vitro*. Therefore, dietary COPs and CHO may influence the oxidation of unsaturated fatty acids resulting in an increase in atherosclerotic lesions. In the present study, dietary COPs, compared to dietary CHO, strongly influenced the fatty acid composition of RBCs. Thus, oxidized CHO may influence fatty acid oxidation and the formation of atherosclerotic lesions. Our unpublished study also showed that dietary oxidized CHO increased the oxidation of unsaturated fatty acid and free fatty acid levels in rabbits. Staprans *et al.* [23] reported that oxidized CHO is absorbed and contributes to the pool of oxidized lipids in circulating lipoproteins. When rabbits were fed oxidized CHO, the fatty streak lesions in the aorta were increased by 100% [23]. They hypothesized that diet-derived oxidized fatty acids in chylomicron remnants, and oxidized cholesterol in remnants and LDL, accelerate atherosclerosis by increasing oxidized lipid levels in circulating LDL and chylomicron remnants. In this regard, we assume that the increases in unsaturated fatty acid levels are influenced to a greater extent by dietary COPs compared to CHO, which contributes to the oxidation of CHO and atherosclerosis in rabbits. This is another possible mechanism of the association of dietary COPs and high natural CHO levels with the increased risk of atherosclerosis in rabbits.

## 4. Experimental Section

### 4.1. Animal Diets and Experimental Protocol

In Study 1, 40 young male New Zealand White rabbits (mean weight, 3 kg) were divided into 5 groups. Each group was balanced with respect to body weight by a restricted randomization technique and individually housed in stainless steel cages during the 3-month feeding trial. After 1 week of acclimation, each group of rabbits was fed a commercial rabbit chow containing 0 g CHO + 0 g COPs/kg diet, 1 g CHO/kg diet, 0.9 g CHO + 0.1 g COPs, 0.8 g CHO + 0.2 g COPs, or 0.5 g CHO + 0.5 g COPs/kg diet. Diets were prepared at 2-week intervals and stored in a cold room (0~5 °C) after vacuum packaging (1 bag per day). Blood samples were collected from ear veins every 2 weeks starting at day 0 of the feeding trial. Four rabbits per treatment were sacrificed by a pentobarbital overdose (200 mg/kg body weight) at days 45 and 90 of feeding. After blood sampling, the thorax was opened, and artery samples were collected.

In Study 2, 24 young male New Zealand White rabbits (mean weight, 3 kg) were divided into 3 groups and fed a chow containing 2 g CHO/kg diet, 1.6 g CHO + 0.4 g COPs, or 1.2 g CHO + 0.8 g COPs. The feeding trial was continued for 12 weeks, and samples were collected as in Study 1. Rabbits fed chow containing 0 g CHO + 0 g COPs/kg diet served as a negative control for both Studies 1 and 2. The feeding, sample collection, and euthanasia protocols were approved by the animal Care Committee of Iowa State University and complied with the Care and the Use of Laboratory Animals. The experimental design is presented in Table 7. The nutrient content of the basal diet in this study is shown in Table 8.

### 4.2. Histopathology

At necropsy, samples of the coronary artery and the ascending thoracic aorta were fixed in 10% neutral buffered formalin for histopathological examination. Tissues were routinely processed and embedded in paraffin wax, and 5–6 μm sections were cut and stained with hematoxylin and eosin (HE) for microscopic examination.

### 4.3. Analyses of Cholesterols, Triglycerides and Lipoprotein in Serum

Serum cholesterols (Kit No. 352 to 20 by Sigma-Aldrich Chemical Co., St Louis, MO, USA) and triglycerides (Kit No. 339 to 20 by Sigma-Aldrich Chemical Co., St Louis, MO, USA) were determined by enzymatic assay kits as specified by the manufacturers.

### 4.4. Analysis of Fatty Acids Composition in Erythrocytes

Blood was collected every two weeks and immediately centrifuged. Erythrocytes were collected and homogenized by adding approximately 10 volumes of deionized distilled water and then centrifuged at 2000× *g* for 15 min. The precipitant was washed and centrifuged until colorless ghost erythrocytes were obtained. Lipids were extracted from the ghost red cells with Folch solution and then dried. One ml of hexane and 1 mL of methylating reagent were added to the 100 μL of lipid extract (from blood cell) and incubated in 90 °C water bath for 1 h. After cooling to room temperature, 2 mL hexane and 5 mL water were added, mixed thoroughly, and left at room temperature overnight for phase separation. The top hexane layer containing methylated fatty acids was analyzed for fatty acid composition using a GC (HP 6890, Hewlett Packard Co., Palo Alto, CA, USA). A ramped oven temperature condition (180 °C for 2.5 min, increased to 230 °C at 2.5 °C/min, then held at 230 °C for 7.5 min) was used. Temperatures of both the inlet and detector were 280 °C. Helium was the carrier gas at linear flow of 1.1 mL/min. Detector (FID) air, H_2_, and make-up gas (He) flows were 350, 35, and 43 mL/min, respectively. Fatty acids were identified by comparison of retention times to known standards. Relative quantities were expressed as weight percent of total fatty acids.

### 4.5. Statistical Analysis

Data were analyzed using SAS software (SAS Inst. Inc., Cary, NC, USA, 2001) by the generalized linear model procedure. The Student-Newman-Keuls’ multiple range test was used to compare differences among means. Mean values, standard deviation of mean (STD), and standard error of mean (SEM) were reported. Significance was defined at *p <* 0.05.

## 5. Conclusions

Atherosclerotic lesions were observed in rabbits only when the levels of dietary CHO and CHO plus COPs were high (2 g total CHO). Dietary CHO and COPs changed lipid metabolism and hyperlipidemic conditions and caused atherosclerosis in the rabbits. Dietary COPs and CHO were thus found to be atherogenic. Further research is needed to determine the effects of dietary CHO on lipid metabolism in the liver and their relationship with the development of atherosclerosis and CHD in animal models.

## Figures and Tables

**Table 1:** Atherosclerotic lesions in ascending thoracic aorta and coronary artery of individual rabbits after feeding diets containing different amounts of cholesterol (CHO) and cholesterol oxides (COPs) for 6 or 12 weeks.

Dietary CHO + COPs g/kg diet	6 weeks		12 weeks	
	
	ATA *	CA	ATA	CA
**Study 1**				

0 g CHO	0	0	0	0
	0	0	0	0
	0	0	0	0

1 g CHO	+	+	+	+
	+	+	+	+
	+	+	+	+
	+	+	+	+

0.9 CHO + 0.1 COPs	+	+	+	+
	+	+	+	+
	+	+	+	+
	+	+	+	+

0.8 CHO + 0.2 COPs	+	+	+	+
	+	+	+	+
	+	+	+	+
	+	+	+	+

0.5 CHO + 0.5 COPs	+	+	+	+
	+	+	+	+
	+	+	+	+
	+	+	+	+

**Study 2**				

2 g CHO	++	+++	+++	++
	+	+	++	++
	++	++	+++	++
	++	++	++	+

1.6 CHO + 0.4 COPs	++++	++++	++++	++++
	+++	++	++	++
	+	+	+++	+++
	+	+	+	+

1.2 CHO + 0.8 COPs	+++	+	+	+
	++	+	++	+
	+	+	+++	++
	+	+	+	+

0, no abnormality detected; +, focal aggregation of 4–8 foam cells; ++, multifocal aggregates of foam cells and lipid; +++, focally extensive thick layers of lipid and foam cells in the tunica intima extending part way around blood vessels; ++++, diffuse thick layer of lipids and foam cells infiltrating the tunica intima and extending around the circumference of the blood vessel and bulging into the lumen;

* ATA, ascending thoracic aorta; CA, coronary artery.

**Table 2:** Effect of dietary cholesterol and cholesterol oxides on total cholesterol content in serum.

Dietary	Feeding periods (weeks)						

Chol * + COPs g/kg diet	0	2	4	6	8	10	12
	mg/dL						

**Study 1**							
0 g	25.8 ± 3.1 c	26.7 ± 4.8 b,c,^z^	26.2 ± 1.9 b,c,^z^	29.7 ± 3.4 b,z	33.8 ± 4.9 a,b,^y^	39.1 ± 6.4 a,y	38.6 ± 1.0 a,y
1 g CHO	25.8 ± 3.1 c	90.5 ± 12.8 b,y	142.3 ± 14.6 b,x,y	271.2 ± 26.3 a,x	263.7 ± 31.3 a,w	258.8 ± 30.0 a,x	334.7 ± 16.9 a,w
0.9 CHO + 0.1 COPs	25.8 ± 3.1 d	142.5 ± 19.9 c,x	139.7 ± 16.6 c,x,y	301.4 ± 39.7 a,b,^x^	223.8 ± 34.2 b,c,^w^	371.6 ± 27.9 a,w	302.0 ± 20.9 a,b,^w^
0.8 CHO + 0.2 COPs	25.8 ± 3.1 c	72.2 ± 11.9 b,y	67.5 ± 17.8 b,y,z	133.1 ± 19.1 a,y	134.2 ± 25.4 a,x	123.9 ± 16.1 a,y	118.8 ± 23.1 a,x
0.5 CHO + 0.5 COPs	25.8 ± 3.1 c	84.6 ± 11.4 b,y	126.7 ± 18.4 a,b,x,y	161.7 ± 21.5 a,y	138.4 ± 15.11 a,b,^x^	132.2 ± 19.1 a,b,^y^	115.9 ± 20.7 a,b,^x^

**Study 2**							
2 g CHO	26.0 ± 2.7 d	390.6 ± 26.4 c,v	620.8 ± 38.4 b,v	701.9 ± 40.7 b,v	616.2 ± 41.2 b,v	767.2 ± 48.7 a,v	620.8 ± 34.6 b,v
1.6 CHO + 0.4 COPs	26.0 ± 2.7 e	326.1 ± 38.4 d,w	398.2 ± 41.1 d,w	645.1 ± 22.5 b,c,^w^	686.8 ± 35.9 a,b,^v^	748.5 ± 41.6 a,v	580.5 ± 36.2 c,v
1.2 CHO + 0.8 COPs	26.0 ± 2.7 c	145.6 ± 22.2 b,x	180.2 ± 10.8 b,x	276.3 ± 23.3 a,x	278.8 ± 44.1 a,w	277.2 ± 24.5 a,w,x	279.7 ± 14.6 a,w

a,b,c,d,e Different letters within a row are significantly different (*p <* 0.05);

v,w,x,y.z Different letters within a column are significantly different (*p <* 0.05);

* chol, natural cholesterol.

**Table 3:** Effect of dietary cholesterol and cholesterol oxides on triglyceride content in serum.

Dietary	Feeding periods (weeks)						

Chol * + COPs g/kg diet	0	2	4	6	8	10	12
	mg/dL						

**Study 1**							
0 g	10.6 ± 1.5 d	15.8 ± 1.9 c,v,w	22.0 ± 2.5 b,c,^x^	28.8 ± 2.9 b,x	37.3 ± 4.0 a,x	38.0 ± 4.5 a,x	40.5 ± 4.9 a,w
1 g CHO	10.6 ± 1.5 d	19.9 ± 3.0 c,v,w	24.8 ± 3.9 b,c,w,x	29.0 ± 3.0 a,b,^x^	34.7 ± 3.0 a,x	32.9 ± 2.1 a,b,^x^	36.2 ± 3.3 a,w,x
0.9 CHO + 0.1 COPs	10.6 ± 1.5 c	20.8 ± 2.2 b,v,w	34.6 ± 4.2 a,w	35.1 ± 4.7 a,w,x	39.3 ± 2.6 a,x	35.5 ± 2.3 a,x	45.9 ± 3.3 a,w
0.8 CHO + 0.2 COPs	10.6 ± 1.5 c	19.1 ± 3.4 b,v,w	20.7 ± 4.4 a,b,^x^	25.1 ± 2.9 a,x	25.3 ± 2.0 a,x	22.0 ± 3.3 a,b,^y^	25.5 ± 4.1 a,x
0.5 CHO + 0.5 COPs	10.6 ± 1.5 c	21.8 ± 3.5 b,v,w	26.8 ± 4.3 a,b,w,x	27.9 ± 2.2 a,b,^x^	24.5 ± 2.5 a,x	29.1 ± 3.9 a,b,x,y	33.2 ± 2.6 a,w,x

**Study 2**							
2 g CHO	11.1 ± 2.1 d	20.0 ± 3.2 c,v,w	54.7 ± 6.3 b,v	63.2 ± 8.9 b,v	65.0 ± 2.6 b,v	69.9 ± 5.1 a,v	77.5 ± 6.5 a,v
1.6 CHO + 0.4 COPs	11.1 ± 2.1 c	30.6 ± 5.9 b,v	27.4 ± 4.9 b,w,x	45.5 ± 6.2 a,b,^w^	49.8 ± 3.3 a,b,^w^	58.6 ± 4.4 a,b,^w^	70.9 ± 7.8 a,v
1.2 CHO + 0.8 COPs	11.1 ± 2.1 e	13.0 ± 1.1 d,w	22.6 ± 2.3 c,d,^x^	24.1 ± 2.9 c,x	28.3 ± 3.7 b,c,^x^	35.3 ± 5.4 bx	46.4 ± 4.9 a,w

a,b,c,d,e Different letters within a row are significantly different (*p <* 0.05);

v,w,x,y.z Different letters within a column are significantly different (*p <* 0.05);

* chol, natural cholesterol.

**Table 4:** Effect of dietary cholesterol and cholesterol oxides on LDL-cholesterol content in serum.

Dietary	Feeding periods (weeks)						

Chol * + COPs g/kg diet	0	2	4	6	8	10	12
	mg/dL						

**Study 1**							
0 g	8.9 ± 0.9 b	8.6 ± 0.7 b,z	8.3 ± 0.8 b,z	9.5 ± 1.1 a,b,^x^	11.2 ± 0.8 a,z	11.4 ± 1.5 a,y	10.4 ± 1.1 a,z
1 g CHO	8.9 ± 0.9 e	73.5 ± 4.5 d,x	124.9 ± 5.7 c,w,x	250.7 ± 13.6 b,v	243.0 ± 6.6 b,c,^w^	237.2 ± 3.2 b,c,^w^	304.1 ± 11.5 a,w
0.9 CHO + 0.1 COPs	8.9 ± 0.9 e	123.3 ± 3.8 d,w	121.3 ± 7.8 d,w,x	279.7 ± 9.5 b,v	202.3 ± 10.2 c,x	348.9 ± 12.6 a,v	270.5 ± 15.8 b,x
0.8 CHO + 0.2 COPs	8.9 ± 0.9 d	54.8 ± 5.1 c,y	48.9 ± 2.4 y,c	114.8 ± 2.3 a,w	114.5 ± 5.9 a,y	103.6 ± 10.0 b,x	93.2 ± 4.3 b,y
0.5 CHO + 0.5 COPs	8.9 ± 0.9 e	66.3 ± 2.7 d,x	108.2 ± 3.0 b,x	143.1 ± 5.9 a,w	116.6 ± 6.4 b,y	110.6 ± 9.2 b,x	90.3 ± 4.7 c,y

**Study 2**							
2 g CHO	8.7 ± 1.4 e	374.2 ± 6.8 d,u	591.6 ± 12.1 c,u	672.6 ± 20.7 b,u	587.5 ± 18.4 c,v	736.9 ± 23.0 a,u	591.8 ± 14.8 c,u
1.6 CHO + 0.4 COPs	8.7 ± 1.4 e	310.9 ± 5.0 d,v	375.7 ± 9.6 d,v	627.9 ± 27.1 b,u	665.6 ± 14.3 b,u	725.1 ± 30.1 a,u	552.5 ± 16.4 c,v
1.2 CHO + 0.8 COPs	8.7 ± 1.4 d	127.3 ± 3.8 c,w	156.9 ± 6.8 b,w	252.5 ± 12.3 a,v	255.2 ± 14.7 a,w	250.3 ± 12.0 a,w	251.7 ± 5.7 a,x

a,b,c,d,e Different letters within a row are significantly different (*p <* 0.05);

u,v,w,x,y.z Different letters within a column are significantly different (*p <* 0.05);

* chol, natural cholesterol.

**Table 5:** Effect of dietary cholesterol and cholesterol oxides on HDL-cholesterol content in serum.

Dietary	Feeding periods (weeks)						

Chol * + COPs g/kg diet	0	2	4	6	8	10	12
	mg/dL						

**Study 1**							
0 g	9.1 ± 1.2 d	18.1 ± 2.3 c	18.9 ± 3.2 c,w,x	20.2 ± 2.9 c,w,x	22.7 ± 3.9 b,c	27.7 ± 3.2 a,b	28.2 ± 1.6 a
1 g CHO	9.1 ± 1.2 c	17.0 ± 5.8 b	17.5 ± 3.1 b,x	20.5 ± 1.4 b,w,x	20.7 ± 1.7 b	21.6 ± 1.7 b	30.6 ± 1.3 a
0.9 CHO + 0.1 COPs	9.1 ± 1.2 d	19.2 ± 1.2 b,c	18.3 ± 1.6 c,w,x	21.7 ± 3.9 b,c,w,x	21.5 ± 2.6 b,c	22.7 ± 2.1 b	30.6 ± 1.3 a
0.8 CHO + 0.2 COPs	9.1 ± 1.2 c	17.5 ± 3.1 b	18.6 ± 1.7 b,w,x	18.3 ± 0.6 b,w,x	19.7 ± 3.1 b	20.3 ± 1.1 b	25.6 ± 4.2 a
0.5 CHO + 0.5 COPs	9.1 ± 1.2 c	18.3 ± 3.8 b	18.6 ± 2.3 b,w,x	18.6 ± 3.2 b,w,x	21.8 ± 3.5 a,b	21.6 ± 2.3 a,b	25.6 ± 3.5 a

**Study 2**							
2 g CHO	9.6 ± 1.9 c	16.4 ± 5.1 b	29.2 ± l.5 a,v	29.3 ± 1.1 a,v	28.7 ± 2.1 a	30.2 ± 2.4 a	29.1 ± 1.8 a
1.6 CHO + 0.4 COPs	9.6 ± 1.9 d	15.2 ± 3.4 c	22.5 ± 2.6 a,b,w,x	17.2 ± 3.0 b,x	21.2 ± 1.4 a,b	23.3 ± 1.9 a,b	28.0 ± 1.9 a
1.2 CHO + 0.8 COPs	9.6 ± 1.9 d	18.4 ± k.3 c	23.3 ± 3.0 a,b,^w^	23.8 ± 3.8 a,b,^w^	23.6 ± 1.1 a,b	26.9 ± 3.3 a,b	28.0 ± 3.7 a

a,b,c,d,e Different letters within a row are significantly different (*p <* 0.05);

v,w,x,y.z Different letters within a column are significantly different (*p <* 0.05);

* chol, natural cholesterol.

**Table 6:** Fatty acid composition of erythrocytes membrane.

	Study 1					Study 2			
	
	0.9 g CHO + 0.8 g CHO 0.5 g CHO +					1.6 g CHO + 1.2 g CHO +			
		
Fatty acid	0 g CHO	1 g CHO	0.1 g COPs	0.2 g COPs	0.5 g COPs	2 g CHO	0.4 g COPs	0.8 g COPs	SEM
	
	Fatty acid (%)								
Myristic acid	3.37 a	3.19 a	3.15 a	3.53 a	3.48 a	0.88 b	0.90 b	1.02 b	0.23
Palmitoleic acid	1.26 b	1.79 a,b	1.77 a,b	1.47 b	1.58 b	1.33 b	2.48 a	1.59 b	0.26
Palmitic acid	36.51 a,b	36.53 a,b	34.51 b	36.81 a,b	36.48 a,b	37.87 a	27.18 c	28.78 c	1.01
Margaric acid	0.89 a,b	0.77 b	1.10 a	0.86 a,b	0.93 a,b	0.31 c	0.39 c	0.31 c	0.10
Linoleic acid	8.53 c	10.14 c	9.76 c	8.19 c	7.47 c	13.91 b	26.39 a	28.00 a	1.25
Oleic acid	5.94 b	8.41 b	10.24 b	7.17 b	8.58 b	18.86 a	18.99 a	18.02 a	2.09
Linolenic acid	0.58 c	0.54 c	0.76 c	1.02 b,c	1.08 b,c	1.68 b	2.68 a	2.10 a,b	0.40
Stearic acid	38.89 a	34.88 a	34.46 a	37.12 a	36.78 a	21.71 b	18.64 b	17.93 b	1.49
Arachidonic acid	2.20 a,b	2.00 a,b	2.35 a	2.08 a,b	1.45 b,c	1.59 b	0.93 c	0.93 c	0.25
Unidentified	1.85 a	1.76 a	1.92 a	1.75^a^	1.81 a	1.88 a	1.44 b	1.39 b	0.09
SFA/USFA	80.2/19.8	75.9/24.1	73.5/26.5	78.8/21.2	78.5/21.5	61.6/38.4	47.4/52.6	48.3/51.7	

a,b,c Different letters within a row are significantly different (*p <* 0.05); *N* = 4.

**Table 7:** Experimental design and amount of cholesterol and cholesterol oxides.

Studies	Treatments	Contents (g)	

		Cholesterol	Cholesterol oxide
Study 1	Control	0	0
	T 1	1	0
	T 2	0.9	0.1
	T 3	0.8	0.2
	T 4	0.5	0.5

Study 2	T 5	2	0
	T 6	1.6	0.4
	T 7	1.2	0.8

**Table 8:** Nutrient content of basal diet rabbit chow.

Nutrient	Content (%)
Crude protein (Min.)	16.0
Crude fat (Min.)	1.5
Crude fiber (Min.)	17.0
Calcium (Min.–Max.)	20.0
Phosphorous (Min.)	0.6–1.1
Salt (NaCl) (Min.–Max.)	0.5–1.0
Vitamin A (Min.)	4,400 IU/kg

Ingredients: processed grain by-products, forage products, roughage products, plant protein products, grain products, molasses products, calcium carbonate, salt, ferrous oxide, dl-methionine, choline chloride, vitamin E supplement, calcium pantothenate, vitamin B-12 supplement, niacin supplement, vitamin A supplement, manganese sulfate, vitamin D-3 supplement, ferrous sulfate, cobalt carbonate, calcium iodate, copper sulfate, zinc sulfate, magnesium oxide, sodium selenite.
